# An Immortal Hero, an Enduring Challenge

**DOI:** 10.3201/eid2207.AC2207

**Published:** 2016-07

**Authors:** Byron Breedlove, Paul M. Arguin

**Affiliations:** Centers for Disease Control and Prevention, Atlanta, Georgia, USA

**Keywords:** art science connection, emerging infectious diseases, art and medicine, about the cover, Hercules and the Erymanthian boar, an immortal hero, an enduring challenge, public health, zoonoses, zoonotic diseases

**Figure:**
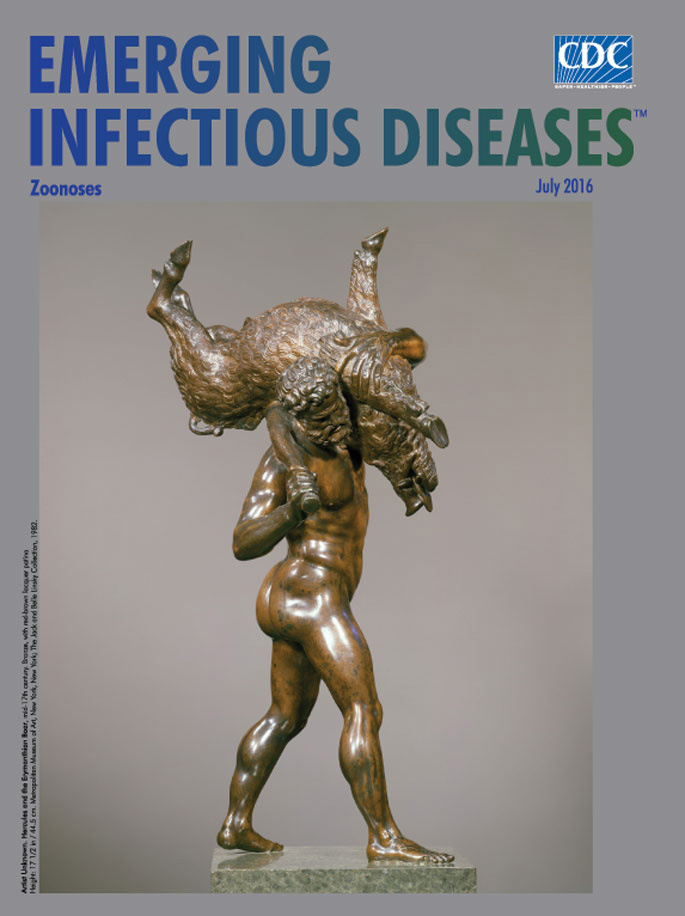
**Artist Unknown. Hercules and the Erymanthian Boar, mid-17th century. Bronze, with red-brown lacquer patina. Height: 17 1/2 in/44.5 cm.** Metropolitan Museum of Art, New York, New York, USA; The Jack and Belle Linsky Collection, 1982.

Hercules^1^ has endured as perhaps the most popular figure from Greek mythology for nearly 3 millennia. Central to his myth is the story cycle about the 12 presumably impossible labors he carried out for the loathed King Eurystheus. Sometimes overlooked is that Hercules performed those tasks as penance for having murdered his own wife and children during a fit of madness.

For the fourth labor, Eurystheus ordered Hercules to capture the vicious Erymanthian Boar, a menacing beast that would descend from its lair on the mountain of Erymanthus each day, trampling the farmlands and attacking man and beast. Surprising the boar in its lair, Hercules drove his quarry into the deep snow, where he subdued the exhausted boar and then carried it to the king’s court. According to the myth, when the cowardly King Eurystheus heard the snorting, grunting beast and realized that Hercules had succeeded, the king hastily hid in a buried pithos jar, imploring Hercules to remove the boar.

Hercules’ capture of the Erymanthian Boar, a tempting subject for artists, has been immortalized on coins and amphora; on a Roman sarcophagus; in paintings, films, and sketches; and through myriad sculptures. The identity of the sculptor who created the elegant bronze statue reproduced for his month’s cover is not known. This work is thought to be modeled on earlier works by an imitator or student of the Flemish sculptor Giambologna (1529–1608), the court sculptor to the Medici grand dukes in Florence. He had produced a set of bronze statuettes of the 12 labors and supervised the work and training of numerous assistants.

This bronze portrays the aftermath of the chase. The figure of Hercules resolutely strides toward the court, clasping the huge boar over his shoulder with his left arm, balancing it with the club he holds aloft in his right hand. The textures of the bronze offer visual and tactile contrasts. Hercules’ body is cast in smooth bronze, which heightens his physical prowess and musculature. The struggling boar’s bristly hide, Hercules’ hair and beard, and the patterns in his wooden club reveal the artist’s nimble touch in working with bronze. A Metropolitan Museum of Art commentary notes that “The present statuette is extremely light in weight, with a dark but warm brown patina and richly variegated tool marks, such as the punch marks that articulate the club.”

Although his strength and vigor are realized through this statue, Hercules would not have redeemed himself through his struggles and suffering alone, and ultimately become immortal, without also using his cunning and skill. He frequently sought the counsel of others in the course of his undertakings. During this labor, it was the centaur Chiron (though some versions say it was a different centaur named Pholus) who advised Hercules to drive the boar into the snow.

While completing his fourth labor, Hercules was potentially exposed to dangers that also threatened mortal men of his time and ours: possible exposure to zoonotic diseases. Zoonotic diseases are caused by any of more than 200 pathogenic agents, including bacteria, viruses, parasites, and fungi, transmitted directly or indirectly from animals to humans. For instance, the Erymanthian Boar could have felled Hercules via microbiology instead of muscle and tusk.

Hercules’ risks did not stop with the Boar. In slaying and then skinning the formidable Nemean Lion and stalking and capturing the sacred Hind of Ceryneia, Hercules was at risk for various zoonotic diseases. Cleaning the Augean stables exposed him to enteric pathogens from the vast quantities of manure generated by teeming herds of cows, sheep, horses, and goats. Additional exposures occurred when he wrestled the Cretan Bull and stole the Cattle of Geryon. Hercules’ efforts to dispel the Stymphalian Birds and capture the Mares of Diomedes may have exposed him to zoonoses acquired from birds and equids. And finally, why was the great 3-headed dog Cerberus so furious and out of control—rabies perhaps?

The human web of daily activities crosses many ecosystems. Animals provide food, transportation, companionship, and if you are Hercules, occasionally a wrestling adversary. Managing and limiting infectious risks associated with the intricate connections among humans, animals, and our environments prove to be challenging, complex endeavors. It’s not hyperbole to label this undertaking a Herculean task.

## References

[1] Gibbons MW. Giambologna: narrator of the Catholic reformation [cited 2016 May 9]. http://ark.cdlib.org/ark:/13030/ft9n39p3vz/

[2] Metropolitan Museum of Art. Hercules and the Erymanthian Boar [cited 2016 Apr 18]. http://www.metmuseum.org/art/collection/search/207005

[3] Tufts Digital Library. The Erymanthian Boar [cited 2016 Apr 23]. http://www.perseus.tufts.edu/Herakles/boar.html

[4] The Walters Art Museum. Hercules carrying the Erymanthian Boar [cited 2016 May 9]. http://art.thewalters.org/detail/8509/hercules-carrying-the-erymanthian-boar/

[5] World Health Organization. Zoonoses and the human-animal-ecosystems interface [cited 2016 May 19]. http://www.who.int/zoonoses/en/

